# Chimeric DNA Vaccines against ErbB2^+^ Carcinomas: From Mice to Humans

**DOI:** 10.3390/cancers3033225

**Published:** 2011-08-10

**Authors:** Elena Quaglino, Federica Riccardo, Marco Macagno, Silvio Bandini, Rodica Cojoca, Elisabetta Ercole, Augusto Amici, Federica Cavallo

**Affiliations:** 1 Molecular Biotechnology Center, Department of Clinical and Biological Sciences, University of Turin, 10126 Turin, Italy; E-Mails: elena.quaglino@unito.it (E.Q.); federica.riccardo@unito.it (F.R.); marco.macagno@unito.it (M.M.); silvio.bandini@unito.it (S.B.); rodica73@libero.it (R.C.); elisabetta.ercole@unito.it (E.E.); 2 Department of Molecular Cellular and Animal Biology, University of Camerino, 62032 Camerino, Italy; E-Mail: augusto.amici@unicam.it

**Keywords:** ErbB2, DNA vaccines, oncoantigens

## Abstract

DNA vaccination exploits a relatively simple and flexible technique to generate an immune response against microbial and tumor-associated antigens (TAAs). Its effectiveness is enhanced by the application of an electrical shock in the area of plasmid injection (electroporation). In our studies we exploited a sophisticated electroporation device approved for clinical use (Cliniporator, IGEA, Carpi, Italy). As the target antigen is an additional factor that dramatically modulates the efficacy of a vaccine, we selected ErbB2 receptor as a target since it is an ideal oncoantigen. It is overexpressed on the cell membrane by several carcinomas for which it plays an essential role in driving their progression. Most oncoantigens are self-tolerated molecules. To circumvent immune tolerance we generated two plasmids (RHuT and HuRT) coding for chimeric rat/human ErbB2 proteins. Their immunogenicity was compared in wild type mice naturally tolerant for mouse ErbB2, and in transgenic mice that are also tolerant for rat or human ErbB2. In several of these mice, RHuT and HuRT elicited a stronger anti-tumor response than plasmids coding for fully human or fully rat ErbB2. The ability of heterologous moiety to blunt immune tolerance could be exploited to elicit a significant immune response in patients. A clinical trial to delay the recurrence of ErbB2^+^ carcinomas of the oral cavity, oropharynx and hypopharynx is awaiting the approval of the Italian authorities.

## 1. Introduction

Despite their broad application, conventional cancer treatments often prolong patient's survival for only a limited period of time, and are associated with devastating side effects. Several new sophisticated approaches are thus being pursued. A multifactorial set of events enables a tumor to grow and metastasize. Several hallmarks of this process have been characterized: sustaining proliferative signaling, evading growth suppressors, resisting cell death, enabling replicative immortality, inducing angiogenesis, activating invasion and metastasis [1]. Additional hallmarks of cells becoming able to give rise to a tumor have recently been defined, such as the ability to modify, or reprogram, cellular metabolism in order to most effectively support neoplastic proliferation, and the ability to evade immune attack by T and B cells, macrophages, and natural killer cells [2].

As to the ability of cancer cells to evade the immune system, the knowledge gained in the last 10 years offers the opportunity to reverse the situation in favor of the immune system and, eventually, the patient. This new information could be channeled to address what seem to be the three major hallmarks for the immune control of cancer progression: Characterization of not-disposable oncoantigens, effective procedures to activate immune reactivity, and strategies to counteract immune suppression [3].

Significant progress in understanding the molecular events involved in the development of an immune response has made specific active immunotherapy a promising anti-cancer strategy. Harnessing adaptive immunity is a powerful tool to treat cancer owing to both the specificity of the immune response elicited, and the possibility of establishing a long-lasting memory [4]. Active immunization's long history of success as a mean of protection against infectious microbial and viral disease has encouraged the search for similar success through the application of vaccination in cancer immunotherapy [5]. Several vaccines modalities have been investigated: protein/peptide vaccines [6-9], *ex vivo* loaded dendritic cells [10-12], recombinant viral/bacterial vectors [13-17] and DNA vaccines [18-22].

DNA vaccination has been shown to possess a number of advantages. It is a relatively simple and very flexible way of activating both the humoral and the cellular immune response in animal models [21,23-25]. Once inside a cell, DNA plasmids encode a protein antigen that is processed as endogenous protein and whose peptides are presented on major histocompatibility complex (MHC) class I molecules on the cell surface. In addition, the protein antigen may be released from transfected cells and thus captured and processed by professional antigen-presenting-cells. In this way, a DNA vaccine activates cytotoxic T cells, T helper cells and antibody responses [26]. Moreover, the plasmid DNA is more than just blueprint for the protein antigen [27] since non-coding hypomethylation dinucleotide cytosine-phosphate-guanine sequences, typical of prokaryotic genome, act as vaccine-embedded adjuvants [28] and trigger the cells of innate immunity [29,30]. These capabilities make DNA vaccines attractive for cancer immunotherapy, though their intrinsically poor immunogenicity in humans remains a major drawback. Several efforts are being made to enhance their potency by exploiting innovative delivery systems. In this review we will summarize our strategy to improve the efficacy of a DNA vaccine in preclinical models based on both transplantable ErbB2^+^ tumors and cancer-prone ErbB2 transgenic mice.

## 2. The Choice of the Target Antigen: The Oncoantigens

The first issue in the development of an effective anti-tumor DNA vaccine is to identify the “best” target antigen. Several efforts have been made to define the ideal features of a tumor associated antigens (TAA) to be used for DNA vaccination. We coined the term “oncoantigens” for TAA that drive the progression of a neoplastic lesion from one stage to the next. Oncoantigens can be expressed on the membrane or in the cytoplasm of a tumor's own cells, or be secreted by the non-neoplastic cells that form the tumor microenvironment [31].

We have combined the employment of transgenic animal models, high throughput technologies, and human data mining to set-up a pipeline for identification of the “best” oncoantigens to be used as targets for cancer vaccines. Comparison of the transcriptome from different stages of tumour progression in cancer-prone transgenic mice revealed oncoantigens with a critical role in these stages. Those identified in a mouse model acquire, however, a special interest when there are equivalents with a corresponding role in human cancer. For this reason, we studied only oncoantigens with a low expression in normal human tissues and a high, homogeneous expression in human cancers. This combination of mouse and human data can be applied to study different kinds of tumors and provide the groundwork for the rational design of distinct cancer vaccines [32].

As our first target we chose ErbB2 receptor. This has the makings of an “ideal” oncoantigen insofar as it plays key roles in numerous physiological processes such as embryogenesis, proliferation, differentiation, adhesion and cell motility, while in adult life it is expressed at low levels and by few cells. By contrast, its overexpression and dysregulation occur in 15% of invasive breast cancers, 54–100% of colorectal cancers, 25% of ovarian cancers, 17–82% of pancreatic cancers and 34% of prostate cancers. These aberrations are associated with greater tumor aggressiveness, increased risk of recurrence, and poor prognosis [33]. In addition, its expression on the cell surface of tumor cells makes ErbB2 a target for both antibodies and cell-mediated immunity.

## 3. Significant Study Models: Mice with a Range of Immune Tolerance to ErbB2

Preclinical trials on cohorts of wild-type mice challenged with transplantable syngeneic ErbB2^+^ tumors or mice engineered to develop a specific ErbB2^+^ carcinomas are used to assess the protective potential of a vaccine and tease apart the mechanisms on which it depends. A vaccine that reduces onset or size of a tumor, or increases survival may be considered for a clinical trial.

By contrast with its mouse ortholog, the rat ErbB2 oncogene has been more widely studied [34]. Most experimental studies are thus carried out on wild-type mice immunized against the rat ErbB2 and challenged with rat ErbB2^+^ mouse tumor cells. In these mice, anti-rat ErbB2 vaccines elicit strong humoral and cellular responses that may lead to spectacular regressions of large tumors expressing the rat ErbB2 [17,35-37]. However, models of this kind are not fully syngeneic since the homology between rat and mouse ErbB2, while very high (95% at amino acid level), is not complete. Thus a powerful immune response against not-tolerate epitopes of a xenogeneic (rat) ErbB2 protein could be mistaken for an anti-tumor response. Moreover, these transplantable tumors do not build up the tumor microenvironment that characterizes spontaneous tumors, while their rapid-growth kinetics minimizes the consequences of tumor genetic instability, immune editing and escape ability of the tumor [38-40]. These tumor-challenge experiments are performed in young and healthy mice whose immune system is much more reactive than that of mice slowly imprinted by an autochthonous tumor [41]. Because of these differences from naturally occurring tumors, the immune response elicited in mouse models of this kind is no more than a starting point for an initial gross validation of the efficacy of an anti ErbB2 vaccine.

Cancer-prone mice transgenic for the rat or human ErbB2 provide more significant models, since their development of an autochthonous tumor recapitulates several of the molecular and genetic features of the progression of human cancer: slow progression, the natural occurrence of invasion and metastasis, and the presence of a long-lasting interaction between the evolving tumor and the host immune system [26].

Expression of the ErbB2 transgene is influenced by its promoter. Even so, it elicits an immune tolerance that may be analogous to that of ErbB2-cancer patients [42]. Rat ErbB2 transgenic mice acquire a central tolerance to rat ErbB2 because its early expression in the thymus causes a central deletion of T cell clones reacting at high affinity with dominant rat ErbB2 peptides [43,44]. Mice transgenic for the human ErbB2, on the other hand, are fully tolerant to human ErbB2 [45]. Since immune tolerance to tumor-associated self-antigens poses a major obstacle to the mounting of an effective response to oncoantigens, these mice transgenic for the rat or human ErbB2 offer an unprecedented opportunity to evaluate the efficacy of a vaccine in breaking tolerance to mouse, rat and human ErbB2.

We have thus been able to assess the efficacy of DNA vaccines against rat and human ErbB2 by challenging wild-type mice naturally tolerant to mouse but not to rat and human ErbB2 with syngeneic tumor cells overexpressing rat or human ErbB2, by challenging mice transgenic and tolerant for rat [34,46,47] and human [45] ErbB2 with syngeneic tumor cells overexpressing rat or human ErbB2, and by evaluating the incidence and development of autochthonous mammary carcinomas in mice transgenic and tolerant for rat and human ErbB2. In both kinds of transgenic mice, mammary carcinogenesis displays a consistent stepwise, age-related progression that mimics several features of human breast carcinoma [34,48]. ErbB2^+^ mammary tumors progress slowly and give rise to spontaneous metastases [48,49]. This slow progression is accompanied by a progressive expansion of both T regulatory cells [50] and myeloid-derived suppressor cells [51] that further hamper the induction of an effective immune response.

Induction of a specific and effective immune response to ErbB2 thus requires the circumvention of both central [43] and peripheral, tumor-induced [44,50] tolerance mechanisms. Spontaneous induction of ErbB2-specific helper and cytotoxic T-cells and serum antibodies in patients with ErbB2^+^ carcinomas [52,53] suggest that these may be possible. The results obtained with the ErbB2 models could then be applied to other oncoantigens.

## 4. A Way to Enhance Anti-ErbB2 Response: The Chimeric Plasmids

**Figure 1.**
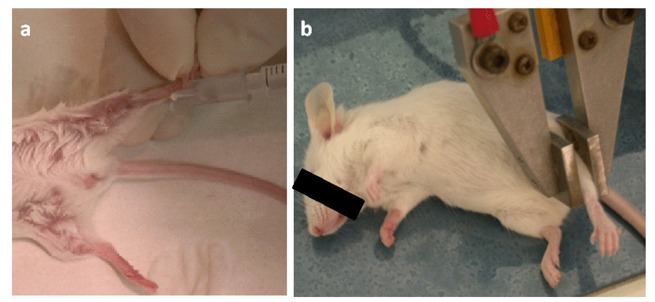
Electro gene transfer performed with the T820 electroporator (BTX, San Diego, CA, USA). **(a)** 25 μg of plasmids diluted in 20 μL of 0.9% NaCl with 6 mg/mL polyglutamate were injected in each of the tibial muscles of anesthetized mice; **(b)** Two square-wave 25 ms, 375 V/cm pulses generated by the T820 electroporator were applied by two electrodes placed on the shaved skin.

To enhance the uptake of DNA plasmids by cells and significantly increase gene expression and the immunogenicity of DNA vaccines [54-56], we combined intramuscular injection of plasmids with *in vivo* electroporation (EP) at first with the T820 electroporator (BTX, San Diego, CA, USA; Figure 1).

Electroporation with DNA plasmids coding for the extracellular and transmembrane domain of the rat (RRT plasmid) or human (HuHuT plasmid) ErbB2 protein elicits a protective response against both rat and human ErbB2^+^ transplantable mammary tumors in wild-type mice. In rat ErbB2 tolerant mice, the RRT plasmid is efficacious against both rat ErbB2^+^ transplantable and autochthonous mammary tumors, but is devoid of any significant effect against human ErbB2^+^ transplantable tumors. Similarly, when human ErbB2 tolerant mice are vaccinated with HuHuT plasmid, the growth of human ErbB2^+^ transplantable human (but not rat) ErbB2^+^ tumor is impaired [24] (Table 1).

Numerous data in the literature show that vaccination with a xenogeneic antigen significantly homologous with the self ortholog is an effective way of overcoming the immunological tolerance to self proteins [57-59]. The immune reaction to xenogenic determinants may be of high affinity, whereas the cross-reactive response to self-homolog antigens may be of lower affinity. Because rat and human extracellular and transmembrane domains of ErbB2 display 84% amino acid homology, one way of enhancing the immune response against ErbB2 was to vaccinate rat ErbB2 transgenic mice with the HuHuT plasmid or human ErbB2 transgenic mice with the RRT plasmid. The vaccines induce a high level of antibodies with a consummate specificity to the ErbB2 ortholog used as immunogen [24,60]. The immune response elicited to human ErbB2 in rat ErbB2 transgenic mice is effective against tumor expressing human ErbB2, but poorly protective against those expressing the rat ErbB2 ortholog. The same is also true when the immune response is elicited against rat ErbB2 in human ErbB2 transgenic mice [24,25]. This ErbB2 ortholog restriction is evident in the immune response elicited by the vaccines to both challenges with syngeneic tumor cells expressing the distinct ErbB2 ortholog, and autochthonous mammary cancer, at least for those appearing in rat ErbB2 transgenic mice [24], since RRT plasmid was not used in human ErbB2 transgenic mice developing autochthonous tumors (Table 1). The strong immunogenic ability of plasmids coding for xenogenic ErbB2 orthologs associated with the poor cross-reaction between these orthologs spurred us to investigate whether vaccination with plasmids coding for chimeric rat/human and human/rat extracellular and transmembrane domains of ErbB2 protein (RHuT and HuRT plasmids) were most effective in blunting immune tolerance to both rat and human ErbB2, and able to elicit a significant cross-reaction.

RHuT encodes a protein in which the 410 NH_2_-terminal residues are from the rat domain and the remaining residues from the human domain; almost symmetrically, HuRT encodes a protein in which 390 NH_2_-terminal residues are from the human domain and the remainder from the rat domain.

Both in wild type mice and in transgenic mice tolerant to rat ErbB2 the highest anti rat ErbB2 antibody titre is induced by vaccination with RHuT, while HuRT is the most effective in inducing an immune response against human ErbB2 in both wild type and human ErbB2 transgenic mice [24,25]. In rat ErbB2 tolerant mice the immunity induced by RHuT confers full protection against a challenge of tumor cells expressing rat ErbB2, and is also the most effective against the onset of mammary carcinomas driven by the expression of rat ErbB2 [24]. In human ErbB2 tolerant mice, while HuRT is clearly the most effective in hampering the growth of transplantable tumors expressing the human ErbB2 ortholog [25], it was equally effective as RHuT in halting human ErbB2^+^ autochthonous mammary carcinomas (Table 2) [24].

Taken together these data suggest that the presence of a heterologous region in the vaccine enhances immunogenicity against a self tolerated ErbB2. Thus, the syngeneic motif of the sequence encoded by the plasmid guarantees the specificity of the induced immune response, while the xenogenic portion ensures better suppression of tolerance.

However, our data suggest that the induced immune response critically depends on the location of this moiety on the chimeric molecule and the different degree of tolerance of the host markedly modulates the induced immune response [24]. In principle, this strategy of combining heterologous with self antigen can be applied to any heterologous TAA that share high level of sequence identity and T cell epitopes to produce a potent DNA vaccine.

## 5. Translational Studies: Preclinical Data for the Design of a Clinical Trial Using the Chimeric Plasmid RHuT

In a pilot clinical trial performed at the Oncology clinic, Radiumhemmet, (Karolinska University Hospital, Stockholm, Sweden), intramuscolar vaccination with a plasmid DNA encoding for a full-length human ErbB2 protein together with low doses of GM-CSF and IL-2 in patients with metastatic breast carcinoma receiving concomitant trastuzumab treatment was shown to be safe and well tolerated. It also induced both specific endogenous antibody responses and late-onset CD4^+^ T-cell responses [61].

The major obstacle to the commercial success of DNA vaccines is undoubtedly their delivery. If this cannot be made simple, cheap and effective, they may not become a viable option for human use. Numerous clinical trials have confirmed that a standard needle and syringe delivery is not enough [62]. An important tenet, confirmed by our DNA immunization studies, is that electroporation not only dramatically enhances the expression of the encoded antigen as well as the potency and immunogenicity of DNA vaccines, but may also enhance the efficacy of DNA vaccines in preventing and/or curing mammary cancer expressing human ErbB2 [21,26].

Devices ensuring safe, tolerable, reproducible and clinically acceptable administration have made electroporation-based DNA vaccination a clinical reality [63]. Electroporation itself can easily be handled as an out-patient procedure, since only local anaesthesia is needed to decrease pain associated with insertion of the needles and the electric pulses [64,65]. In this regard, electrochemotherapy and electro-gene transfer with the Cliniporator™ (IGEA Srl, Carpi, Italy) has been added to clinical practice. Electrochemotherapy is currently proposed for the treatment of relapsed/refractory cutaneous melanoma patients [66-68]. It is a safe procedure, easily performed in terms of toxicities and cost effectiveness ratios, that combines chemotherapy and electroporation to increase drug uptake, and is hence a more efficient way of enabling non-permeant or poorly permeant chemotherapeutic agent to enter tumor cells than simple injection into the tumor [64,69]. No human electroporation-based gene transfer trials have been completed, though several are under way [65].

Our studies on ErbB2 transgenic mice have shown that progression of neoplastic lesions stemming from an ErbB2 gene alteration can be more effectively counteract by: (i) DNA plasmid electroporation as compared to intramuscolar vaccination; (ii) the adoption of chimeric rat/human plasmids instead of fully homologous or fully xenogeneic plasmids [21,24].

**Figure 2.**
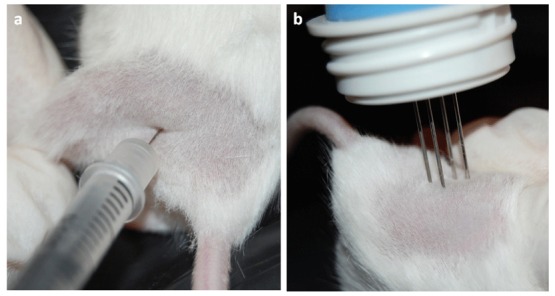
Electro gene transfer performed with the Cliniporator™ (IGEA Srl, Carpi, Italy). **(a)** 25 μg of plasmids diluted in 20 μL of saline were injected into the quadriceps muscle of anesthetized mice; **(b)** Immediately after the injection two electric pulses consisting of a low-voltage pulse of 100 V and of 200-ms duration were applied by an array needle electrode inserted in the injected area.

These data would seem to justify the undertaking of a clinical trial of electroporation of the RHuT plasmid in patients with ErbB2^+^ carcinomas. As a step in this direction, we have tried out our DNA vaccination protocols by using the Cliniporator™, a device approved for clinical use. Mice were anesthetized and injected in the quadriceps muscle with 50 μg of plasmid DNA diluted in 20 μL of saline. This was immediately followed by insertion of an array needle electrode [70] in the injection site to apply two square-wave pulses of 25 ms in length and 110 V in intensity (Figure 2). Each course of vaccination consisted of two DNA injections followed by electroporation with an interval of 14 days.

**Figure 3.**
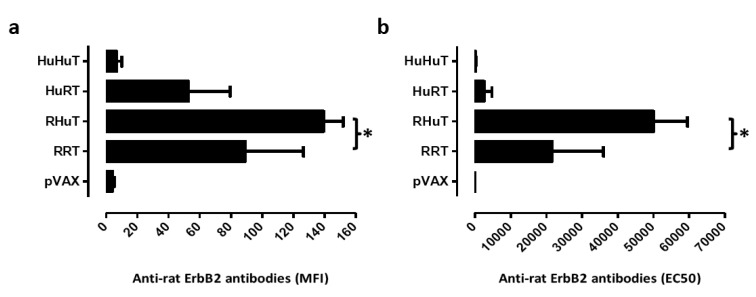
Antibody response against rat ErbB2 protein two weeks after vaccination of BALB/c mice with pVAX, RRT, RHuT, HuRT and HuHuT. The anti-rat ErbB2 antibody titre was evaluated both by flow cytometry (a) and by ELISA (b). **(a)** Sera were incubated with rat ErbB2^+^ and rat ErbB2^-^ fibroblasts and anti-mouse IgG antibodies as previously described [21]. Data are reported as Mean Fluorescence Intensity (mean MFI ± SEM). *, p = 0,02, Student's t test; **(b)** The anti-rat ErbB2 antibody titre was detected by indirect ELISA and is expressed as EC50 (mean EC50 ± SEM) *, p = 0,03, Student's t test. Rat ErbB2 protein (0.1 μg/well in PBS pH 7.4) was incubated at 4 °C overnight, and then washed two times with PBS plus 0.05% Tween 20 (PBST). Antigen coated wells were blocked using 1% BSA at 37 °C for 2 hr and washed twice with PBST. Serum samples were added, and the plate was incubated at 37 °C for 2 hr, washed two times with PBST and supplemented with goat-anti mouse IgG HRP conjugate in PBST-BSA. After 90 min incubation at 37 °C, the wells were washed with PBST and 100 μL of ready prepared substrate solution (TMB) were added and incubated for 15 min at RT. The reaction was stopped and the absorbance was measured at a wavelength of 450 nm.

Since enhancement of antibody levels is the most evident immune response to RRT and RHuT plasmids induced with the T820 electroporator [24,25], two weeks after the second vaccination the ability of vaccination with the Cliniporator™ to induce an effective humoral immune response was evaluated. Analysis of the sera of wild type vaccinated BALB/c mice showed that RHuT induced a significant higher anti rat ErbB2 antibody titre than RRT. Similar results were observed when this titre was evaluated by flow cytometry (Figure 3a) and by ELISA (Figure 3b).

**Figure 4.**
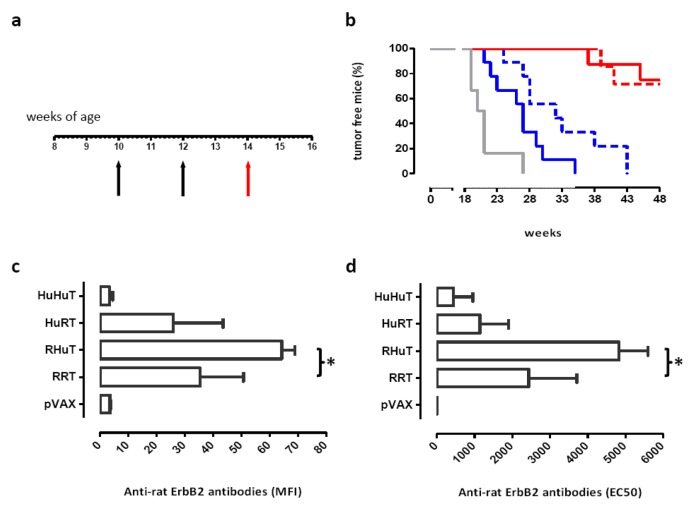
Protection against autochthonous ErbB2^+^ mammary tumors provided by vaccination with RRT, RHuT, HuRT and HuHuT plasmids in BALB-neuT mice and anti rat ErbB2 antibodies induction. **(a)** Experimental protocol: BALB-neuT mice were vaccinated with different plasmids when they displayed mammary atypical hyperplasia (black arrows). Two weeks after the second vaccination they were bled (red arrow), sera were collected and stored for anti rat ErbB2 antibodies evaluation; **(b)** Mammary tumor incidence of BALB-neuT vaccinated mice with RRT (dotted red line, n = 7 mice), RHuT (continuous red line, n = 8 mice), HuRT (dotted black line, n = 9 mice), HuHuT (continuous black line, n = 9 mice) and empty control pVAX (dotted grey line, n = 6 mice). Differences in tumor incidence were analyzed by the log-rank (Mantel-Cox) test. The anti-rat ErbB2 antibody titre was evaluated both by flow cytometry **(c)** and by indirect ELISA procedure **(d)**. Data are reported as mean MFI ± SEM (* p = 0,02, Student's t test) and as mean EC50 ± SEM (* p = 0,04, Student's t test).

The effect of this vaccination on autochthonous tumors was also investigated. Ten-week-old BALB-neuT mice were vaccinated when their mammary glands displayed atypical hyperplasia (Figure 4a). HuHuT and HuRT significantly delayed mammary tumor incidence, but offered no protection against the onset of rat ErbB2^+^ mammary tumors. By contrast, RRT and RHuT significantly extended the tumor-free survival since 72% of RRT- and 75% of RHuT-vaccinated mice were tumor-free until week 48, at which time all HuRT- and HuHuT-vaccinated mice displayed at least one palpable tumor (Figure 4b). Indeed, induction of antibodies against rat ErbB2 correlates with the protection induced by vaccination: the highest titre is associated with the best protection (Figures 4c and 4d).

These results lay the foundation for the phase I/II clinical trial proposed by our laboratory (EudraCt number: 2005-001432-74). This is a single centre, prospective, adjuvant, trial in patients treated by surgical resection and/or chemotherapy and/or radiotherapy for primary, locally advanced (III and IV stages), human ErbB2^+^ carcinomas of the oral cavity, oropharynx, hypopharynx. Its primary objective is to assess the feasibility and toxicity of DNA vaccination against human ErbB2 with the RHuT plasmid. The secondary objective is to determine whether such vaccination results in an immune response to human ErbB2.

## 6. Conclusions: Road Map to the Design of a Potentially Successful anti-Cancer Vaccine

**Figure 5.**
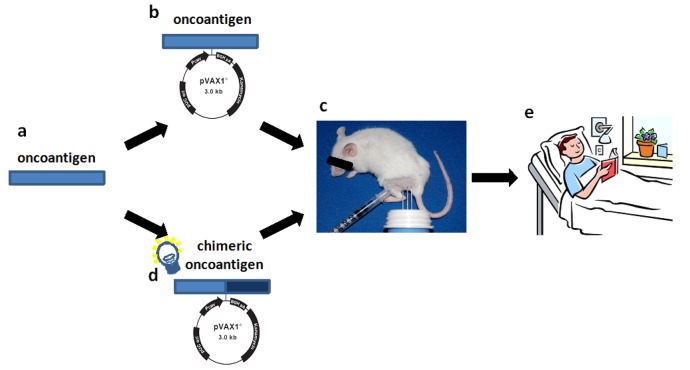
Road map for the design of an effective strategy to develop an efficacious anti tumor DNA vaccine to be used in cancer patients. **(a)** The oncoantigen to use for active immunization should be identified from tumors arising in cancer prone transgenic mice as suggested by the pipeline proposed by Cavallo *et al.* [32]; **(b)** The efficacy of a DNA plasmid coding for the entire or part of the oncoantigen identified should be tested in tolerant transgenic mice; **(c)** Insertion of heterologous sequences coding for the oncoantigen inside the plasmid DNA vaccine (chimeric plasmid) should be built up to enhance the immunogenicity of the vaccine; **(d)** The protective immune response elicited by chimeric plasmid should be evaluated in tolerant transgenic mice of different genetic background, if possible; **(e)** Induction of an effective anti tumor immune reaction in these mice lay the basis for the design of a clinical trial.

Promising preclinical results indicate that cancer immune prevention could be applied to humans to reduce the risk of cancer [5]. Early data from clinical trials with DNA vaccines delivered by electroporation are cautiously promising. Thus, we may be entering a new era of DNA vaccination where we start to see clinical effects in humans. But, there is still much to do in terms of optimizing vaccine design and way of administration. Our findings might be useful guides for the translation of a vaccine to clinical practice since theoretically, the strategy used to enhance the immunogenicity and anti-tumor efficacy of ErbB2 vaccine may be applicable to other tolerated oncoantigens (Figure 5).

All these studies deal with ErbB2, the most promising archetypal oncoantigen. However, it is important to explore whether other oncoantigens targeted by vaccination may provide similar or complementary protection. The use of cancer-prone mice engineered to carry defined genetic alterations relevant to human cancer has led us to the definition of a pipeline for the identification of oncoantigens to be used as vaccination targets [32]. Since several mouse models of autochthonous tumours that mimic crucial features of human cancer exist, a list of new oncoantigens that function at specific stages of carcinogenesis could be obtained [71] (Figure 5a). As oncoantigens are self molecules, the ability of the vaccine coding for the oncoantigen sequence to induce an effective immune response should be tested in transgenic tolerant mice (Figure 5b). Even if tolerance to an oncoantigen in mice may be very different from that in humans, the information acquired could provide an initial starting point for the development of an effective antitumor vaccine. A new vaccination regimen consisting of vaccines coding for heterologous sequences and self-sequences of the oncoantigen could provide a more potent DNA vaccine (Figure 5c). However, since immune reaction to heterologous determinant may induce low-affinity cross-reactive response to self-homolog antigens, the efficacy of the chimeric vaccine should be tested again in transgenic tolerant mice (Figure 5d). However, the different genetic makeup and the different state of tolerance to the oncoantigen of patients could represent a critical issue for the induction of an effective antitumor immune reaction. For this reason, for future vaccine design, the efficacy of chimeric vaccine should be tested in transgenic mice of different genetic background. The generation of a robust B-cell and T-cell response to the self-antigen together with a measurable anti-tumor effect in transgenic tolerant mice will provide the basis for the use of the chimeric vaccine to treat cancer patients, and will hold the promise of an interesting clinical perspective (Figure 5e).

## Figures and Tables

**Table 1.** ErbB2 orthologue restriction of the protection against tumor growth elicited by anti-ErbB2 DNA vaccines.

**Immunizing plasmid**	**Recipient mouse**	**Challenging Tumor**		**Autochthonous Carcinoma**	
	
		**ErbB2 orthologue expressed**	**Efficacy of the protection**	**ErbB2 orthologue expressed**	**Efficacy of the protection**
RRT	Wild-type	rat	+++*	NA#	NA
		human	+++	NA	NA
	Transgenic for rat ErbB2	rat	++	rat	++
		human	-	NA	NA
	Transgenic for human ErbB2	rat	+++	NA	NA
		human	-	human	ND$
HuHuT	Wild-type	rat	+++	NA	NA
		human	+++	NA	NA
	Transgenic for rat ErbB2	rat	-	rat	-
		human	ND	NA	NA
	Transgenic for human ErbB2	rat	ND	NA	NA
		human	+++	human	ND$

* -: 0% protection;

+ : from 0% to 30% protection;

++ : from 30% to 70% protection;

+++ : from 70% to 100% protection;

# Not applicable;

$ Not Done

**Table 2.** ErbB2 orthologue restriction of the protection against tumor growth elicited by anti-ErbB2 DNA chimeric vaccines.

**Immunizing plasmid**	**Recipient mouse**	**Challenging Tumor**		**Autochthonous Carcinoma**	
	
		**ErbB2 orthologue expressed**	**Efficacy of the protection**	**ErbB2 orthologue expressed**	**Efficacy of the protection**
RHuT	Wild-type	rat	+++*	NA#	NA
		human	+++	NA	NA
	Transgenic for rat ErbB2	rat	+++	rat	+++
		human	+++	NA	NA
	Transgenic for human ErbB2	rat	+++	NA	NA
		human	++	human	+++
HuRT	Wild-type	rat	+++	NA	NA
		human	+++	NA	NA
	Transgenic for rat ErbB2	rat	-	rat	-
		human	+++	NA	NA
	Transgenic for human ErbB2	rat	++	NA	NA
		human	+++	human	+++

* -: 0% protection;

+ : from 0% to 30% protection;

++ : from 30% to 70% protection;

+++ : from 70% to 100% protection;

# Not applicable
